# Tuning High and Low Temperature Foaming Behavior of Linear and Long-Chain Branched Polypropylene via Partial and Complete Melting

**DOI:** 10.3390/polym14010044

**Published:** 2021-12-23

**Authors:** Mu Sung Kweon, Mahmoud Embabi, Maksim E. Shivokhin, Anvit Gupta, Xuejia Yan, George Pehlert, Patrick C. Lee

**Affiliations:** 1 Multifunctional Composites Manufacturing Laboratory (MCML), Department of Mechanical and Industrial Engineering, University of Toronto, 5 King’s College Road, Toronto, ON M5S 3G8, Canada; mkweon@mie.utoronto.ca (M.S.K.); embabi@mie.utoronto.ca (M.E.); 2 ExxonMobil Chemical Company, 5200 Bayway Drive, Baytown, TX 77520, USA; maksim.e.shivokhin@exxonmobil.com (M.E.S.); anvit.gupta@exxonmobil.com (A.G.); xuejia.yan@exxonmobil.com (X.Y.); george.j.pehlert@exxonmobil.com (G.P.)

**Keywords:** linear and long-chain branched polypropylene, batch foaming, high-pressure differential scanning calorimetry, crystallization onset temperature, extensional hardening, strain hardening ratio

## Abstract

While existing foam studies have identified processing parameters, such as high-pressure drop rate, and engineering measures, such as high melt strength, as key factors for improving foamability, there is a conspicuous absence of studies that directly relate foamability to material properties obtained from fundamental characterization. To bridge this gap, this work presents batch foaming studies on one linear and two long-chain branched polypropylene (PP) resins to investigate how foamability is affected by partial melting (Method 1) and complete melting followed by undercooling (Method 2). At temperatures above the melting point, similar expansion was obtained using both foaming procedures within each resin, while the PP with the highest strain hardening ratio (13) exhibited the highest expansion ratio (45 ± 3). At low temperatures, the foamability of all resins was dramatically improved using Method 2 compared to Method 1, due to access to lower foaming temperatures (<150 °C) near the crystallization onset. Furthermore, Method 2 resulted in a more uniform cellular structure over a wider temperature range (120–170 °C compared to 155–175 °C). Overall, strong extensional hardening and low onset of crystallization were shown to give rise to foamability at high and low temperatures, respectively, suggesting that both characteristics can be appropriately used to tune the foamability of PP in industrial foaming applications.

## 1. Introduction

Polymer foaming is a well-established technology and has involved numerous research efforts that aim to achieve the advantageous properties of cellular plastics, such as their high strength-to-weight ratio, enhanced impact strength, thermal insulation, sound absorption, and lightweight qualities [1,2,3]. These desirable characteristics have allowed polymeric foams to find widespread applications in areas such as building and construction, packaging, automotive, and consumer products. Naturally, the use of polymer foams in a number of different fields has been accompanied by a growing demand for the understanding of foamability and the foaming temperature range of various polymer resins. Due to its ease of implementation, batch foaming technology has been widely employed as an efficient and inexpensive method for studying the foaming behavior of a polymer. Earlier batch foaming studies have investigated commodity plastics, such as polystyrene and polyethylene, because of their versatile nature against other competitive materials; however, these thermoplastics face limitations in their usage due to poor physical properties and low service temperature [4]. To overcome these drawbacks, polypropylene (PP) has been considered as an attractive resin in the foaming industry, owing to its low material cost, low density, high service temperature, and high mechanical strength. Existing literature on PP batch foaming abounds with studies that explore its foaming behavior under the effects of crystallinity [5], blends [6,7,8,9,10,11,12], fillers [13,14,15,16,17,18,19,20], molecular modification [21,22], saturation temperature and pressure [23,24,25], and physical blowing agent [26,27].

In a typical batch foaming process, a polymer sample is placed and sealed in a chamber that is injected with gas to saturation at a certain temperature, pressure, and period of time. This step is followed by gas release that induces a rapid pressure drop in the polymer-gas mixture, which in turn promotes the nucleation of bubbles and their subsequent growth. Cell nucleation predominantly proceeds via heterogenous nucleation, in which impurities, such as catalyst residuals or foreign particles, in the polymer lower the surface energy cost for bubble nuclei formation. In semi-crystalline thermoplastics, pre-existing crystals serve as effective heterogeneous cell nucleation sites during foaming; however, it is more challenging to gain precise control over the foaming behavior of semi-crystalline polymers, owing to their heterogeneous nature, compared to amorphous polymers. In an attempt to study the effect of crystallinity on the morphology of microcellular foam structures, Doroudani et al. revealed that more slowly cooled PP samples, which have higher crystallinity, produced more non-uniform cellular structures [5]. The authors noted that since gas cannot impregnate crystallites, the amorphous regions surrounding the crystalline mass were highly concentrated with gas and often contained small, nucleated cells that contributed to a high cell density at the expense of uniformity of the overall cellular structure.

As such, improving the cell uniformity and foam structure has been a major research direction in the foaming of conventional linear PP, which has a low melt strength that makes cell walls susceptible to rupture during cell growth. Therefore, to achieve improvement in the foamability and cellular structure of linear PP by means of increasing the melt strength, researchers have blended the material with other polymers of higher melt strength or exploited creative techniques, such as molecular modification. Shi et al. investigated different blend compositions of linear isotactic PP (iPP) and a commercial grade of high-melt-strength PP (HMS-PP) to enhance the melt strength and microcellular foamability of iPP under supercritical carbon dioxide (scCO_2_). This attempt successfully showed that introducing 20 wt% of HMS-PP to the iPP matrix can increase the average cell density by an order of magnitude, decrease the average cell size from 174.63 to 2.17 µm, and increase the volume expansion ratio by 126% [11]. On the other hand, Li et al. took a different approach and presented a facile process to introduce ionic interactions to PP to enhance its melt strength. The study reported a series of modified PP ionomers with different strengths of ionic associations that led to different degrees of melt elasticity, demonstrating convenient tunability of rheological properties and foamability [21].

In addition to methods involving chemical alterations to enhance melt strength, strategies investigating the effect of processing parameters on foamability have also been explored. Xu et al. conducted a collective study that investigated the synergistic effects of saturation temperature and pressure on the expansion ratio and cell morphology of PP foams produced using scCO_2_ as the physical blowing agent [23]. The authors reported that a form of “equivalence” was observed between saturation temperature and pressure based on how equal expansion ratios were achieved in foam samples obtained at 154 °C and 25 MPa compared to 157 °C and 16 MPa under the same pressure drop rate. They argued that an increase in temperature was effectively equivalent to an increase in saturation pressure, as an increase in either processing parameter enhanced the mobility of PP chains and the diffusivity of scCO_2_ in the polymer matrix. Chen et al. used a different approach to increase the apparent solubility of scCO_2_ in PP by introducing a pressure swing saturation that led to the dissolution of gas in a shorter amount of time compared to that in a conventional batch foaming process [28]. As a result, this strategy widened the foaming temperature range and increased the expansion ratio, which may also be due to the repeated depressurization steps inducing the nucleation of cells that effectively served as nucleation sites for subsequent bubble nucleation. To take advantage of heterogeneous bubble nucleation and produce low-density foams from neat PP, Fu et al. induced alignment in linear PP by applying planar extensional flow during compression molding prior to foaming [29]. The authors demonstrated that the elongated “brick and mud” crystal structure was effective in producing low-density foams with high compressive strength and thermal stability.

A common aspect shared by most existing batch foaming studies using scCO_2_ is that foaming is initiated under conditions where the polymer is partially molten to determine the optimal foaming behavior, while practical foaming applications involve complete melting of thermoplastics for ease of processability under applied flow, such as in extrusion foaming and foam injection molding. Some batch foaming studies have attempted to explore the sole effect of temperature on foamability by saturating PP at elevated temperatures and cooling to lower temperatures for foaming [28,29,30,31]; however, gas saturation was performed over several hours, which realistically may lead to crystallization of the polymer matrix prior to foaming, given such prolonged durations. To bridge this gap in understanding the foamability of PP in its partially molten state versus fully amorphous state as a melt, the work presented here aims to investigate the effect of the saturation heating profile on the final foam structure and foaming window while completing gas saturation in the order of minutes. In this fundamental study, PP foaming was conducted using a batch foaming apparatus, in which the sample undergoes either (1) partial melting during isothermal gas saturation or (2) complete melting by heating of the polymer beyond its melting point to erase all pre-existing crystals, followed by non-isothermal saturation. By incorporating three commercial grades of PP with different molecular structures, this comparative and comprehensive approach not only provides rich insight into how PP starts to foam from a semi-crystalline state compared to a fully molten state, but also reveals how strain hardening enhances foamability, especially at high temperatures. High-pressure differential scanning calorimetry, extensional rheology, and scanning electron microscopy were used as fundamental characterization techniques to identify that lower crystallization onset and a higher degree of extensional hardening are favorable for obtaining foams with uniform cell morphology over a wide temperature window. Moreover, the introduction of this new temperature protocol allows batch foaming to be brought one step closer to industrial foaming processes, thereby establishing greater common grounds between both approaches for better chances of batch foaming being employed as a method for screening the foamability of newly developed resins prior to their application in continuous processes.

## 2. Materials and Methods

### 2.1. Materials

**Figure 1:**
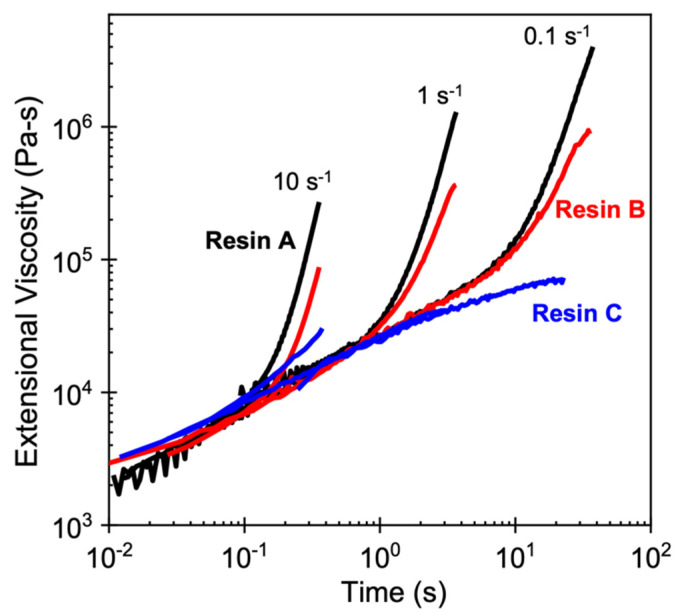
Extensional rheology of the three resins used for foaming studies in this work. Measurements were performed at 190 °C using an ARES-G2 rheometer with SER fixture based on the ExxonMobil test method EM514. SHR values at an extension rate of 1 s^−1^ and a Hencky strain of 3 for Resin A, Resin B, and Resin C are 13, 5.6, and 1.0, respectively.

The materials studied in this work were three different commercial HMS-PP homopolymers. The material parameters as well as the physical and molecular properties of the polymers are presented in Table 1. The major difference across the resins is that Resin A and Resin B are long-chain branched while Resin C is linear in chain architecture. Owing to this difference in molecular structure, the branched polymers exhibited strong extensional hardening that was absent in their linear counterpart (Figure 1). Between the two branched polymers, Resin A showed a stronger strain hardening behavior than Resin B at all tested extension rates. To quantify these differences in extensional rheology, the degree of strain hardening is expressed as a strain hardening ratio (SHR)—the ratio of the transient elongational viscosity ($\eta_{E}^{+}\left(t\right)$) to transient viscosity in the linear viscoelastic regime (3$\eta^{+}\left(t\right)$) at a given strain. In this work, SHR was evaluated at an extension rate of 1 s^−1^ and a Hencky strain of 3. To prepare the test specimens for foaming experiments, pellets were compression molded (Model 4386 Bench Top Laboratory Manual Press, Carver Inc., Wabash, IN, USA) into 5 × 5 × 3 mm^3^ samples for 10 min at 200 °C and 1000 psi and quenched in cold water. CO_2_ (purity > 99.99%) purchased from Messer Group GmbH (Mississauga, ON, Canada)was used as the physical blowing agent.

### 2.2. Batch Foaming Test Protocol

**Figure 2:**
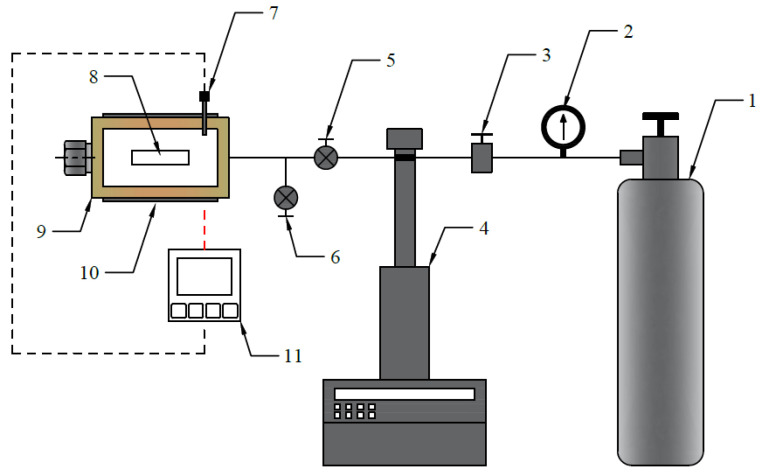
Schematic representation of the batch foaming system: (1) CO_2_ cylinder, (2) pressure gauge, (3) refill valve, (4) syringe pump, (5) inlet valve, (6) exhaust valve, (7) thermocouple, (8) sample, (9) chamber, (10) band heater, and (11) temperature controller.

The foaming experiments in this study were conducted using a small-scale batch foaming apparatus with a chamber where the temperature was accurately controlled by a band heater with proportional-integral-derivative feedback control. As shown in Figure 2, a CO_2_ gas cylinder was connected to the chamber through a pipeline, and a syringe pump (Teledyne ISCO 260D) was used to supply a metered gas stream to maintain the CO_2_ pressure inside the chamber at a constant pressure of 2000 psi.

**Figure 3:**
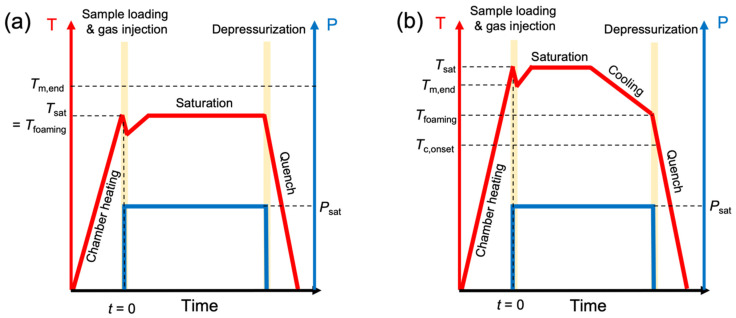
Temperature–pressure profile of foaming method used to investigate the effect of (**a**) partial (Method 1) and (**b**) complete melting (Method 2) on PP foamability. *T*_m,end_, *T*_sat_, and *T*_c,onset_ denote end melting, gas saturation, and onset crystallization temperatures, respectively.

Two different temperature profiles were employed to investigate the effect of partial versus complete melting during the gas saturation stage on the foamability of the PP resins, referred to as “Method 1” and “Method 2” (Figure 3). Method 1 is the approach that is more commonly used in conventional batch foaming studies and involves: (1) pre-heating the chamber to a designated saturation temperature (*T*_sat_); (2) loading the sample and sealing the chamber; (3) injecting CO_2_ into the chamber and allowing the sample to saturate isothermally at the designated temperature; and (4) rapidly depressurizing and quenching the chamber after 18 min of saturation. These steps were repeated over a temperature range of 145–175 °C at 5 °C intervals to explore the temperature(s) at which the PP resins exhibited optimal expansion behavior. In Method 2, a significantly different test protocol was applied to erase pre-existing crystals and thereby induce foaming in a supercooled melt. Foaming using this method involves the following steps: (1) pre-heating the chamber to a fixed temperature of 210 °C; (2) loading the sample and sealing the chamber; (3) injecting CO_2_ into the chamber to saturate the sample at 210 °C for a certain period of time; (4) powering off the heat supply and allowing the chamber to cool down at a constant rate of 5.5 °C/min to reach the designated foaming temperature (*T*_foaming_); and (5) rapidly depressurizing and quenching the chamber after a total saturation time of 18 min. This method was applied over a temperature range of 120–170 °C at 10 °C intervals to explore the expansion behavior of the resins at lower temperatures that were inaccessible in Method 1, as long as the samples remained in their fully molten state. To ensure that the samples in both procedures were subjected to a total saturation time of 18 min, the isothermal saturation duration at 210 °C in Method 2 was adjusted for each foaming temperature, since the cooling rate was fixed at 5.5 °C/min. In other words, the lower the foaming temperature in Method 2, the longer it took for the chamber to reach *T*_foaming_ under a constant cooling rate; therefore, the time spent during isothermal saturation at 210 °C decreased with decreasing foaming temperature to maintain the total saturation time at 18 min.

### 2.3. High-Pressure Differential Scanning Calorimetry

**Figure 4:**
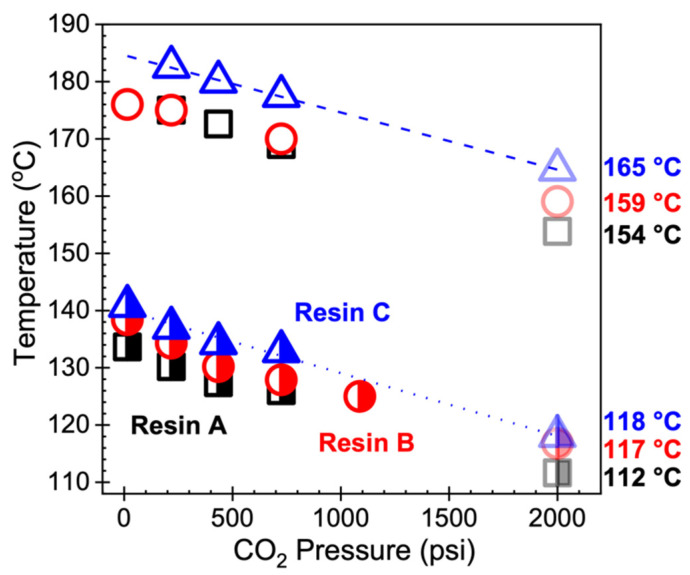
HP-DSC measurements of the end melting point (open) and onset crystallization temperature (half-filled) with respect to CO_2_ pressure. Data points at a CO_2_ pressure of 2000 psi were estimated using linear extrapolation (applicable up to 3600 psi [32] for *T*_c,onset_ (dotted line) and 2000 psi for *T*_m,end_ (dashed line) [33]).

The changes in melting and crystallization temperatures with respect to CO_2_ pressure were determined using a high-pressure differential scanning calorimeter (HP-DSC, DSC 204 HP Phoenix^®^, NETZSCH, Selb, Germany). Samples were placed in an aluminum pan and saturated with CO_2_ at the desired pressure in the HP-DSC chamber. The system was then equilibrated at 50 °C, heated to 210 °C at a rate of 10 °C/min, held isothermal for 5 min, and cooled to 50 °C at a rate of 5.5 °C/min. This temperature profile in the HP-DSC was designed to mimic the thermal history experienced by the sample in the batch foaming chamber under Method 2. The heat–cool cycle was repeated over a range of CO_2_ pressures, from atmospheric pressure to 1000 psi. For each resin, the onset crystallization temperature (*T*_c,onset_) at 2000 psi (i.e., saturation pressure in batch foaming experiments) was estimated by applying linear extrapolation to the measured data (Figure 4), as this assumption has been shown to be valid for CO_2_ pressures up to 3600 psi [32]. Similarly, the end melting temperature (*T*_m,end_) at 2000 psi for each resin was obtained using linear extrapolation (valid up to 2000 psi [33]). The *T*_c,onset_ values of Resins A, B, and C were 112, 117, and 118 °C, respectively; the *T*_m,end_ values were 154, 159, and 165 °C, respectively. Figure 4 shows that the crystallization and melting temperatures decrease linearly with increasing CO_2_ pressure.

### 2.4. Foam Characterization

The foamability of the resins was evaluated according to the expanded volume of the foamed samples. The volume expansion ratio (Φ) was calculated as the ratio of the density of the unfoamed material (0.9 g/cm^3^) to the density of the foamed sample. The latter quantity was obtained using a water displacement method (ASTM D792-13) to compare the mass of the foam in air and in water [34]. Three different samples at a given test condition were used to calculate the average expansion ratio and standard deviation.

Morphological characterization of the cell structure of the polymer foams was performed using a scanning electron microscope (SEM, Generation 5 Phenom Pro, Nanoscience Instruments, Phoenix, AZ, USA). Samples for SEM imaging were prepared by cryogenically fracturing the foamed sample and sputter coating the exposed cross section with a platinum conductive layer. The obtained images were analyzed using ImageJ software to characterize the cell count (*n*) in a given area (*A*), from which the cell density (*N*) was determined:

(1) $$N={\left(\frac{n}{A}\right)}^{3/2}\times \Phi$$

The size of at least 50 cells was also measured to calculate the average cell size and cell size distribution.

## 3. Results

### 3.1. Volume Expansion Ratio

**Figure 5:**
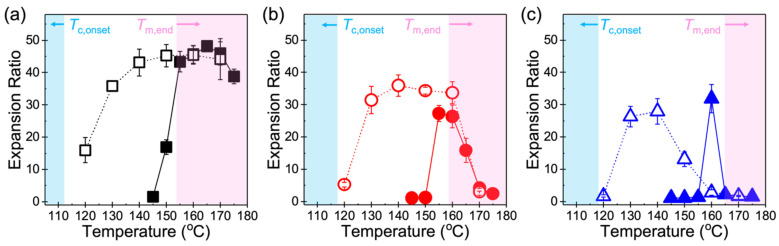
Volume expansion of (**a**) Resin A, (**b**) Resin B, and (**c**) Resin C with respect to foaming temperature. Closed and open symbols (with lines as a guide to the eye) refer to expansion ratios obtained using Method 1 and Method 2, respectively. Shaded areas indicate low and high temperature regions determined by the respective *T*_c,onset_ and *T*_m,end_ values obtained from HP-DSC measurements in Figure 4.

Figure 5 demonstrates that the two foaming methods did not result in any significant difference in the maximum achievable expansion within each resin; however, they led to stark differences in the temperature range over which foaming occurred. In particular, the most apparent effect of partial (Method 1) versus complete melting (Method 2) of PP is reflected in the breadth of the temperature window, where Method 2 resulted in a broader foaming window compared to Method 1. Considering the foaming window as the difference between the maximum and minimum temperatures that produce foams with Φ > 10, Method 2 substantially widened the foaming window by at least 20 °C for all three resins compared to Method 1, resulting in 50 °C, 30 °C, and 20 °C for Resin A, Resin B, and Resin C, respectively. Among the three polymers subjected to either method, Resin A (Figure 5a) exhibited the widest foaming window as well as the highest expansion ratio overall. Between Resin B (Figure 5b) and Resin C (Figure 5c), the former showed a broader window than the latter; however, the maximum expansion achieved was similar. Based on these observations, it can be postulated that the breadth of the foaming window is determined by the chain architecture, namely long-chain branched PP tends to foam over a wider temperature range than linear PP, irrespective of the foaming method.

Considering the different temperature ranges over which foaming occurs, the dramatic improvement in the foamability of a sample subjected to Method 2 as opposed to Method 1 arises from the fact that the expansion behavior under the latter method is complicated by the presence of pre-existing crystallites, while the former method is solely governed by the onset of crystallization in an amorphous melt. Upon complete melting and subsequent cooling of PP to the desired *T*_foaming_ in Method 2, the sample remains in its supercooled state as a polymer–gas mixture as long as foaming is induced above *T*_c,onset_. Thus, high expansion ratios can be achieved over a wide range of temperatures until *T*_foaming_ approaches *T*_c,onset_ (blue shaded regions in Figure 5). A sharp decrease in the expansion ratio was observed in all resins when foaming was induced at the lowest *T*_foaming_ of 120 °C near the onset of crystallization, because a significant reduction in chain mobility may play a dominant role in restricting the growth of nucleated cells. On the other hand, a sample that undergoes Method 1 requires sufficient melting of the polymer or a considerably long saturation time (in the order of hours) for CO_2_ to dissolve in [5,10,35], since the presence of pre-existing crystals gives rise to tortuosity that makes it more challenging for gas molecules to diffuse into the amorphous regions. In particular, the longer tortuous path created by crystalline domains and the reduction of free volume in the amorphous phase may impede diffusion of CO_2_ into PP [36], consequently resulting in poor foamability in samples produced using Method 1 at a *T*_foaming_ far below *T*_m,end_ (red shaded regions in Figure 5). Increasing the temperature to initiate foaming 4–5 °C below *T*_m,end_ led to a noticeable increase in the expansion ratio in all resins, suggesting that the sample in the foaming chamber is primarily in its molten state and contains a small but adequate amount of crystallites that serve as highly effective bubble nucleation sites similar to nucleating agents that are typically added in foaming processes to promote heterogenous cell nucleation [37,38]. When *T*_foaming_ exceeded *T*_m,end_, both methods led to comparable expansion ratios in all three resins. The similarity in the foaming behavior under such high temperature conditions may be attributed to the fact that there is essentially no difference between Method 1 and Method 2 under the same saturation time and pressure when *T*_foaming_ > *T*_m,end_.

### 3.2. Cell Morphology

**Figure 6:**
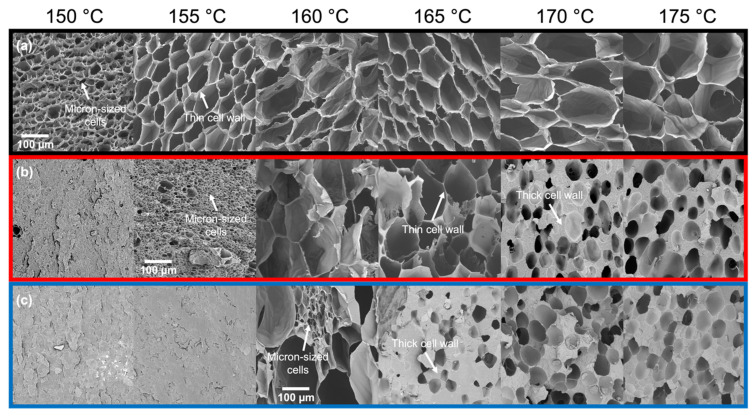
SEM images of (**a**) Resin A, (**b**) Resin B, and (**c**) Resin C foams produced using Method 1 (i.e., partial melting of PP). End melting temperatures of the resins are 154 °C, 159 °C, and 165 °C, respectively.

**Figure 7:**
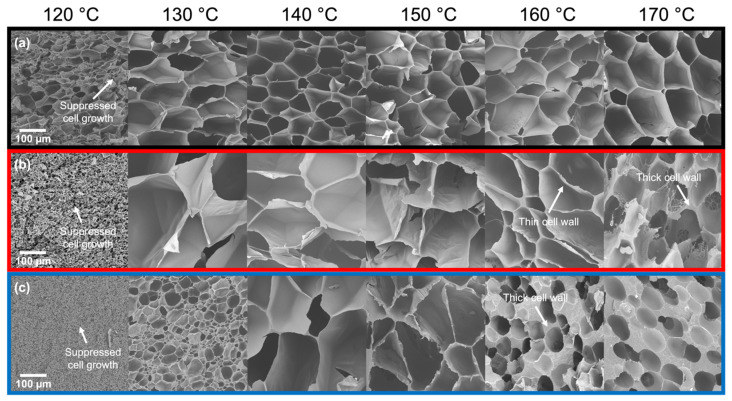
SEM images of (**a**) Resin A, (**b**) Resin B, and (**c**) Resin C foams produced using Method 2 (i.e., complete melting of PP followed by supercooling). Onset crystallization temperatures of the resins are 109 °C, 117 °C, and 118 °C, respectively.

The cell morphologies of the foamed samples are further evidence of how the foaming methods result in similar expansion behavior at high temperatures. In the *T*_foaming_ > *T*_m,end_ regime, Resin A (*T*_m,end_ = 154 °C) generally showed uniform cell structures up to 175 °C in Method 1 (Figure 6a), which are comparable to those in Method 2 (Figure 7a; 160 °C and 170 °C). In the case of Resin B (*T*_m,end_ = 159 °C), foams with thin cell walls were produced from Method 1 under the temperature range of 160–165 °C, beyond which gas loss dominated the foaming process and resulted in small nucleated bubbles segregated by thick cell walls (Figure 6b; 170 °C and 175 °C). This behavior in Resin B was also observed in foams produced using Method 2 (Figure 7b) at similar foaming temperatures above *T*_m,end_, where 160 °C led to a cell morphology characterized by thin cell walls, while 170 °C resulted in thicker cell walls. Such observations are consistent with Resin C, which foamed at or above its end melting temperature of 165 °C using Method 1 (Figure 6c) and at 160–170 °C using Method 2 (Figure 7c), where fast diffusion of CO_2_ at high temperatures allows gas molecules to escape the polymer melt upon pressure release prior to the stabilization of foam structures. Evidence of gas loss can be directly seen in the photographs of samples foamed at elevated temperatures (Figure S1), where concentrated areas of gas pockets were formed on the surface of the foam or ruptured to allow gas to escape.

**Figure 8:**
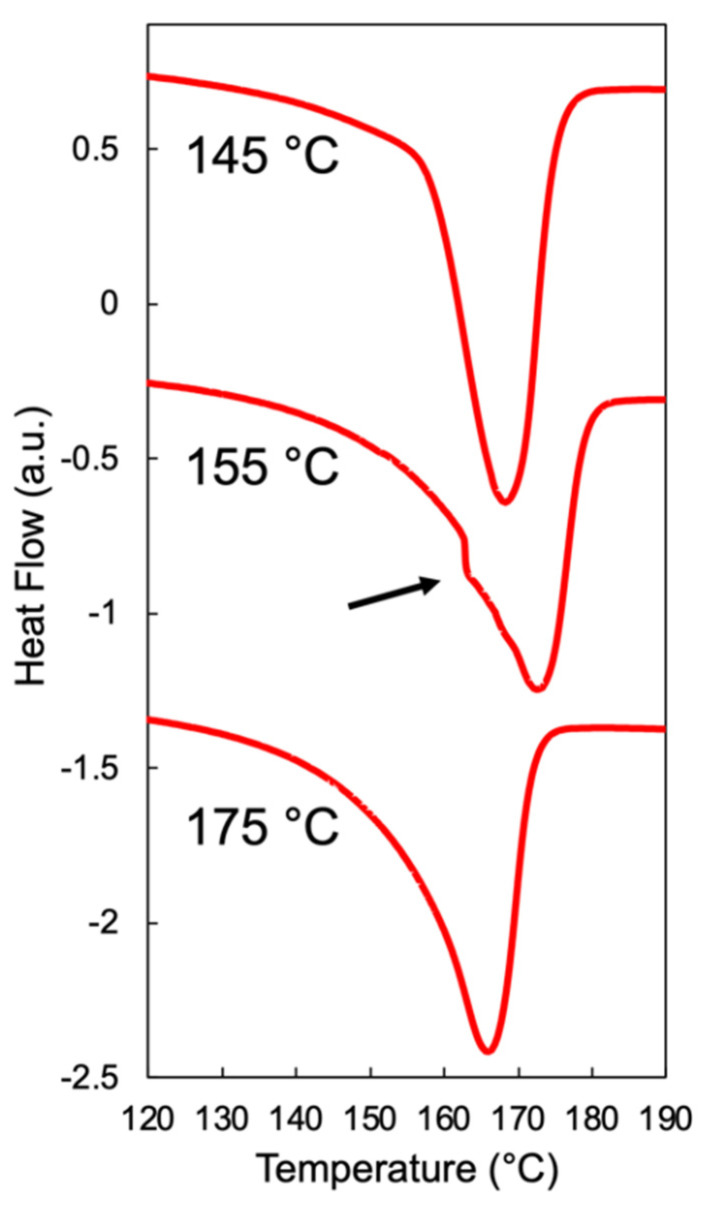
Heating endotherm of Resin B foamed using Method 1 as measured by DSC. Shoulder at 155 °C reflects the presence of unmolten crystallites prior to foaming, and shift in melting peak to a higher temperature indicates heterogenous crystallization onto existing crystals.

When foaming is induced below *T*_m,end_, discrepancies in the cell structure resulting from the two methods begin to emerge. In Method 1, the smallest cell sizes were observed at temperatures 4–5 °C below the *T*_m,end_ of each resin (Figure 6). The corresponding cell morphology was characterized by micron-sized cells for Resin A and Resin B with the exception of Resin C, which showed a bimodal cell size distribution separated by an order of magnitude (Figure S2). Based on these observations, the optimal foaming behavior of each resin in Method 1 may be the result of the aforementioned intricate balance between a small presence of crystalline domains and an abundance of amorphous regions dissolved with CO_2_. This speculation is supported by the differential scanning calorimetry (DSC) measurements of samples foamed at different temperatures under Method 1 (Figure 8). Using Resin B as the representative material, the sample foamed at 155 °C—which led to the optimal expansion ratio and smallest cell size—exhibited a shoulder near the melting peak in the heating endotherm. The appearance of this shoulder confirms that crystallites were present in the sample prior to foaming, and the melting peak at a higher temperature indicates that new crystals were formed during foaming onto the existing crystallites via heterogeneous nucleation. Such behavior was absent in the DSC curves of the samples foamed at the two temperature extremes (145 °C and 175 °C), as reflected in the single melting peak. Hence, it can be deduced that the optimal foaming behavior of resins under Method 1 is governed by the saturation temperature that sufficiently melts the polymer but leaves a small number of crystals unmolten.

**Figure 9:**
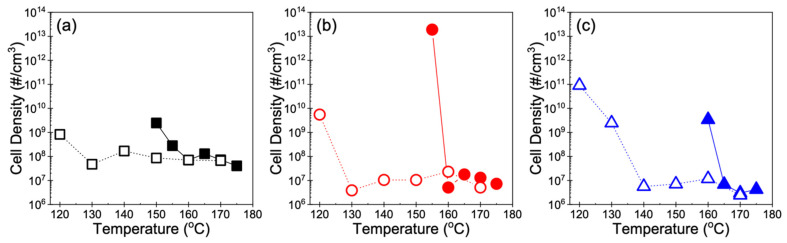
Cell density of (**a**) Resin A, (**b**) Resin B, and (**c**) Resin C foams. Units of cell density correspond to number (#) of nucleated bubbles per volume of foamed polymer. Closed and open symbols (with lines as a guide to the eye) correspond to cell density measurements obtained from foams produced using Method 1 and Method 2, respectively.

**Figure 10:**
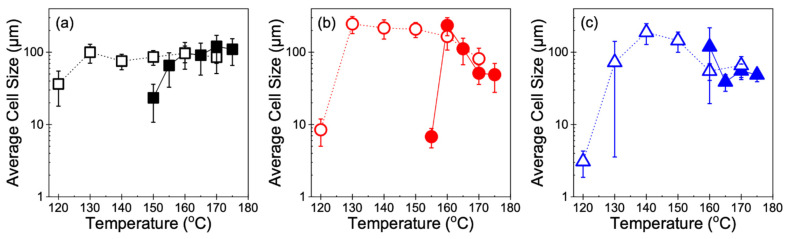
Average cell sizes of (**a**) Resin A, (**b**) Resin B, and (**c**) Resin C foams. Closed and open symbols (with lines as a guide to the eye) correspond to Method 1 and Method 2, respectively.

In addition, the optimal foaming behavior is characterized by a sharp increase in cell density. For all three resins subjected to Method 1, there was an increase of several orders of magnitude in the cell density of foams obtained at the temperature nearest to *T*_m,end_ compared to that of samples obtained at higher temperatures (Figure 9). At temperatures well below *T*_m,end_, particularly in Resin B and Resin C, *T*_foaming_ may be too low to melt the polymer and the saturation time may be too short for sufficient CO_2_ dissolution to occur to allow any cells to nucleate. As such, the high sensitivity of the foaming behavior to temperature limits the cellular structure to remain uniform only over a narrow temperature range in Method 1. However, in Method 2, the SEM images (Figure 7) and cell density measurements (Figure 9) of foamed samples were respectively in qualitative and quantitative agreement across a wide range of temperatures for each resin. At lower temperatures, especially where samples were unable to be foamed using Method 1 (e.g., *T*_foaming_ < 160 °C for Resin C), the resins were not only capable of foaming but continued to show uniform cell structures. Such uniformity in cell morphology that was maintained over a wide temperature range is also reflected in how the cell density generally remained constant throughout most of the foaming temperatures explored in Method 2. When *T*_foaming_ is sufficiently low and approaches *T*_c,onset_, the polymer chains become stiffer and thus may restrict cell growth. As a result, nucleated bubbles grow into fine cells that lead to orders of magnitude increase in cell density despite the low volume expansion. This behavior at the lowest *T*_foaming_ was also revealed in a sharp decrease in the average cell size at the lowest foaming temperature (Figure 10).

## 4. Discussion

The results presented above demonstrate that the foaming of supercooled PP (Method 2) is a simple strategy that is capable of broadening the foaming window without introducing additional parameters to conventional batch foaming processes (Method 1) or modifying the molecular structure of polymers. In fact, the maximum achievable expansion ratio and cell density obtained under both methods were essentially the same in each resin, while there was a distinct difference in the range of temperatures over which foaming occurred (Table 2). Among the resins, Resin A exhibited the highest volume expansion, widest foaming window, and highest cell density. Resin B and Resin C showed similar maximum expansion ratios and a range of cell densities, except that the former PP had a broader foaming window. These findings demonstrate that long-chain branched (LCB) PP foams across a wider range of temperatures compared to linear PP, as mentioned earlier and as suggested in the current literature, which also suggests that PP with a higher melt strength value may be more desirable for foaming. However, while melt strength is a convenient measure that captures information about the resistance of a molten polymer to stretching, it is not quite representative of traditional extensional viscosity measurements. Melt strength is obtained by pulling an extrudate using a variable speed under non-isothermal conditions as it exits a die, which means that the measured force response is complicated by both temperature and deformation rate, in addition to the molecular properties of the polymer itself. Therefore, the governing factors for foamability in polymers are yet to be elucidated from a more fundamental perspective involving material characterization—particularly in terms of which material properties dictate the volume expansion at a given temperature, determine the breadth of the foaming temperature window, and give rise to uniformity in the foam morphology. In light of the overall observations made of the expansion ratios and cell morphologies from the two methods described in this work, we propose that there are two governing factors that capture the foamability of a semi-crystalline polymer in batch foaming: crystallization onset and degree of strain hardening. In particular, it is postulated that (1) a lower onset temperature of crystallization allows PP to foam at lower temperatures and (2) a higher degree of strain hardening allows for foaming at higher temperatures.

### 4.1. Foamability at Low Temperatures: Effect of Crystallization Onset

**Figure 11:**
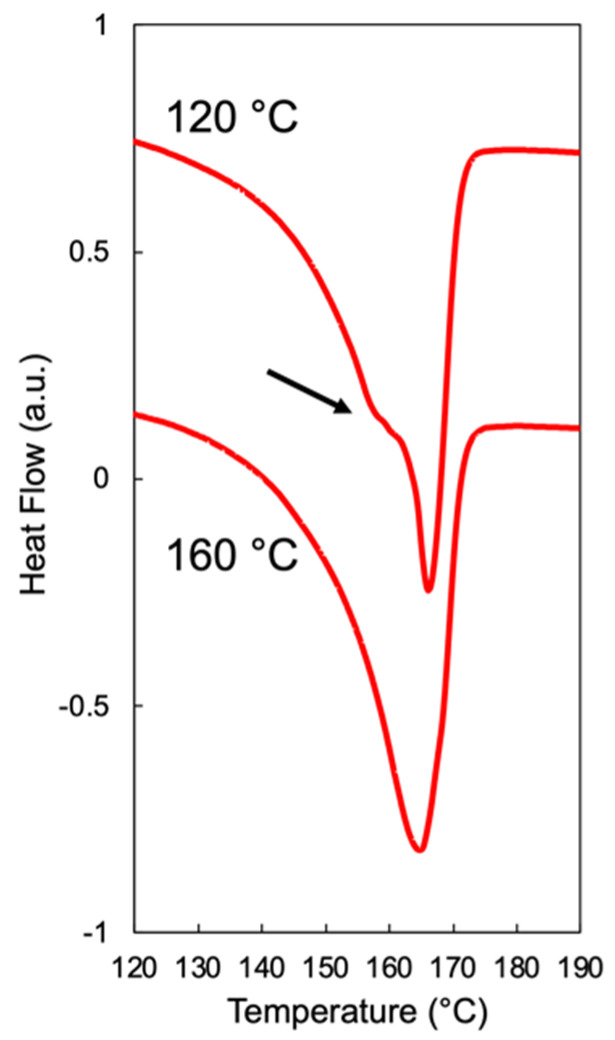
Heating endotherm of Resin B foamed using Method 2 as measured by DSC. Shoulder in the DSC curve of the sample foamed at 120 °C reflects the formation of crystals during cooling.

The effect of crystallization onset on foaming becomes relevant when a semi-crystalline polymer is fully molten and foamed in its supercooled state. Thus, when it comes to the foaming of polymers using Method 2 described in this work, the onset of crystallization can be interpreted as the lower temperature limit above which noticeable volume expansion can be achieved. The question then becomes how far above the *T*_c,onset_ the sample needs to be to allow cell nucleation and sufficient bubble growth, in which the latter may be significantly affected by the viscosity of the polymer melt. That is, if the temperature of the molten polymer is too low, its chain mobility may be restricted and thereby limit the growth of nucleated bubbles. Such behavior was observed in Resins B and C under Method 2 at the lowest foaming temperature of 120 °C, which is 2–3 °C above the *T*_c,onset_ value estimated from HP-DSC data. While cell sizes in the range of 1–10 µm were achieved in samples foamed at this temperature, the expansion ratio below five reflects that the samples could hardly expand. Upon further analysis, the suppressed expansion at the lowest foaming temperature was shown to be the result of crystals developed during cooling and non-isothermal saturation, as reflected in the DSC measurements of Resin B foamed at 120 °C versus 160 °C under Method 2 (Figure 11). In particular, the heating endotherm of Resin B foamed at 160 °C exhibited a single melting peak while that at 120 °C showed a clear shoulder at a lower temperature and a melting peak at a higher temperature, similar to the observations made in Figure 8. While crystallization was not anticipated to occur until 117 °C in Resin B, the fact that crystals were formed at 120 °C implies that either *T*_c,onset_ was underestimated or that there was a mismatch between the temperature of the sample and that of the foaming chamber. The repeatability of the foaming experiments strongly suggests that the temperature control of the system is reliable and accurate; hence, it is more likely that the linear extrapolation resulted in an underestimation of *T*_c,onset_. In fact, foaming experiments at smaller temperature intervals would be able to reveal the degree of underestimation; however, that is beyond the scope of this work. It is more important to realize that the estimation may be a couple degrees off, as reflected in the noticeable expansion achieved in Resin A at 120 °C, which is 8 °C above the perhaps underestimated *T*_c,onset_ of 112 °C. Therefore, it can be concluded that foaming induced at temperatures at least 8 °C above *T*_c,onset_ may lead to sensible expansion of the molten polymer–gas mixture. When the foaming temperature is further above *T*_c,onset_, optimal foaming behavior—characterized by high volume expansion and uniform cell density—is achieved, which often emerges as a “mountain-shaped” profile in continuous foaming processes as the die temperature approaches the crystallization temperature of the polymer–gas mixture [39].

A different way of understanding the effect of crystallization onset on foaming is by considering how much the polymer is supercooled in Method 2. According to the Hoffman nucleation theory for polymer crystallization, the degree of supercooling (ΔT) is defined as the difference between the equilibrium melting temperature ($T_{m}^{0}$) and the crystallization temperature [40]. Several works have estimated the $T_{m}^{0}$ of iPP from the Gibbs–Thompson plot using different techniques, such as optical microscopy [41] and differential scanning calorimetry [42], resulting in a value of 186 °C. In a study on the crystallization kinetics of linear and LCB PP, Tian et al. [43] used the Hoffman–Weeks linear extrapolation method [44] to estimate the $T_{m}^{0}$ of linear PP as 190 °C and that of LCB PP with a high level of branching as 191 °C. Assuming that the three PP resins studied in the present work have comparable $T_{m}^{0}$ values similar to those in the literature, the degree of supercooling in each of the three resins is essentially dictated by their individual onset crystallization temperatures. Therefore, it can be said that PP possessing a higher ΔT is capable of foaming over a wider range of temperatures, particularly due to the low *T*_c,onset_. This premise is consistent with the observations made of the expansion behavior of resins subjected to Method 2, where *T*_c,onset-A_ < *T*_c,onset-B_ < *T*_c,onset-C_ resulted in Φ_A_ > Φ_B_ > Φ_C_ for samples foamed at the lowest *T*_foaming_ of 120 °C (Figure 5). The importance of supercooling becomes more evident when a comparison is made between the foaming behaviors resulting from Method 1 and Method 2. In the former method, the sample never experiences undercooling, unless it undergoes complete melting and subsequent cooling. As such, not only is foaming limited to only a narrow range of temperatures, but also the cellular morphology of foams obtained at low temperatures is extremely non-uniform due to pre-existing crystallites and/or crystals that are newly formed via heterogenous nucleation [5].

### 4.2. Foamability at High Temperatures: Effect of Strain Hardening

At high foaming temperatures above *T*_m,end_, the expansion behavior can be directly explained using the degree of extensional hardening in the polymer. The SHR values of the resins reveal that PP with a higher SHR was capable of foaming to appreciable expansion ratios at higher temperatures (Table 2). It is understood that the relevant extension rates and Hencky strains applied to the polymer melt during the growth of nucleated bubbles are in the order of 1–5 s^−1^ and 3–4, respectively [36]. Hence, given that the melt viscosities of the three polymers were similar (as reflected in the linear viscoelastic region in Figure 1), Resin A was able to tolerate the largest amount of stress induced by cell growth owing to its high SHR value (SHR = 13). In fact, the thin cell walls of Resin A at high temperatures are direct evidence of how strong strain hardening in the polymer melt helps to capture the growing cells and stabilize the cell walls upon cell nucleation induced by pressure release, despite the fact that gas loss is present at such elevated temperatures. Based on this premise, the lower SHR value of Resin B (SHR = 5.6) may make it challenging for the melt to prevent cell wall rupture at high temperatures, as thin cells would fail to maintain their structure under extensional stress upon cell growth. Accordingly, Resin C (SHR = 1.0) was unable to foam at any temperature above *T*_m,end_ due to the absence of strain hardening (Figure 5).

It must be noted that strain hardening behavior and long-chain branching are typically associated with each other, where branched polymers subjected to extensional flow show substantial strain hardening. However, it is critical to note that the converse of this statement is not necessarily true—not all polymer melts that exhibit strain hardening are long-chain branched. Wagner et al. identified that the molecular origin of strain hardening is chain stretching in both linear and branched polyolefins [45]. The authors demonstrated that branched polyolefin melts exhibit more pronounced strain hardening because the molecular stress function, which describes the stress in the polymer chain, shows a quadratic dependence on the applied strain according to the quadratic molecular stress function theory that accurately captures the transient extensional viscosity of long-chain branched polyolefins; in contrast, linear polyolefins that show upturn in the time-dependent elongational viscosity have lower degrees of strain hardening because the linear molecular stress function theory more accurately describes their extensional rheology. Therefore, it is imperative to consider long-chain branching and strain hardening separately when attributing these characteristics to foaming behavior, especially when it comes to comparing linear and branched polymers. In other words, it would be fallacious to conclude that fully molten linear polymers do not foam because of their linear structure; rather, the poor foamability should be attributed to a lack of strain hardening.

This observation then leads to the question of why linear PP foams in its molten state at lower temperatures, as Resin C showed significant foamability at 130–150 °C under Method 2. Noting that Resin C showed weak but unambiguous extensional hardening at 10 s^−1^ (SHR = 1.6; Figure 1), shifting the rheological response at 190 °C to lower temperatures (more relevant to foaming conditions) suggests that the extensional viscosity at these temperatures may be sufficient to prevent cell wall rupture and thus leads to substantial volume expansion. From a rheological perspective, the strength of the flow produced by an extension rate of 10 s^−1^ at 190 °C is equivalent to the flow strength resulting from slower rates at lower temperatures. Using the time–temperature shift factor (*a_T_*) of Resin C obtained from linear viscoelastic characterization, the flow strength of 10 s^−1^ at 190 °C is rheologically equal to those of 2.2 s^−1^ at 130 °C (*a_T_* = 4.5) and 3.8 s^−1^ at 150 °C (*a_T_* = 2.6). Therefore, it can be deduced that the small but distinctly positive upturn in elongational viscosity in Resin C allowed it to reach a sufficiently high extensional viscosity under the relevant extension rates during cell growth at lower temperatures, thereby resulting in foaming.

### 4.3. Other Perspectives on Foamability

Other than molecular attributes of the polymer, crystallization behavior and crystallization kinetics have been suggested as characteristics that control the foaming behavior of PP. Weingart et al. compared the foamability of two different grades of linear HMS-PP and HMS LCB PP (similar to Resin A in this work) in extrusion foaming and identified slow crystallization kinetics and low crystallization temperature of linear PP as the key factors for achieving low density foams with uniform cell morphology [46]. In their work, linear PP with the slow crystallization kinetics and low onset of crystallization showed the best foam quality, whereas the HMS LCB PP produced the poorest foams owing to its fast crystallization kinetics and high crystallization onset, as determined by differential scanning calorimetry and rheometry. Based on their findings, the authors claimed that crystallization behavior was of the utmost importance for good foamability, and questioned whether strain hardening, observed in HMS LCB PP but not in linear PP, can be a reliable measure. However, it must be noted that the resins compared in their work vary in molecular properties. The linear PP that showed the best foamability in extrusion foaming had a weight average molecular weight and polydispersity index that were greater than those of the HMS LCB PP by 120,000 g/mol and by a factor of 2.4, respectively. Furthermore, the onset of crystallization in the rheometer was monitored in the absence of any dissolved gas under conditions far from what the polymer experiences in foam extrusion. As such, a comparison between branched and linear polymers with similar molecular properties and characterization performed under conditions more relevant to those in extrusion foaming may be necessary to demonstrate that strain hardening is not a governing factor in foaming, and thus would require further investigation.

In fact, the current literature abounds with foaming studies that attribute long-chain branching in a polymer to increasing its melt strength [47] or inducing strain hardening behavior [48], thereby improving its foamability. In contrast, a limited number of studies have considered the effect of partial and complete melting on broadening the foaming window of semi-crystalline polymers. Li et al. [49] and Hou et al. [32] remain as the few researchers that have created foams from linear PP in its supercooled state. Li and colleagues investigated methods to foam linear PP without any molecular modifications, and successfully fabricated PP foams with bimodal cell structures. Hou and co-workers advanced this concept and produced open-cell PP foams for oil adsorption applications. However, a direct comparison of the foamability of linear and long-chain branched PP that share similar material properties has not been conducted to date. Rather, existing studies have relied on melt strength or long-chain branching to generally describe their effects on foaming. Ding et al. employed temperature protocols almost identical to those described in the present work to compare the foamability of PP in batch foaming using scCO_2_ as the blowing agent [30]. While they also reported that a broader foaming window was obtained when foaming was initiated on a completely molten PP sample, a saturation time of 2.5 h—compared to 18 min in the present work—was used. Such a prolonged saturation may allow the sample to crystallize isothermally during this time frame, which is likely to result in no effective difference between the two temperature protocols. Furthermore, the authors did not report any DSC measurements on the melting and crystallization temperatures, which makes it rather difficult to investigate the effects of end melting point and crystallization onset temperature, as described in the work presented here.

## 5. Conclusions

To this end, the work presented here offers valuable insights into the two key characteristics that give rise to expansion behavior at high and low temperatures: strong strain hardening under extensional flow and foaming initiated at a supercooled state. The effect of strain hardening is highlighted in the positive correlation between the SHR values and the volume expansion ratios at high temperatures, such as 160 and 170 °C. Compared to the other two resins, Resin A was able to tolerate the largest amount of extensional stress induced by cell growth owing to its high SHR value, which is reflected in its high volume expansion under both methods. The high SHR value also contributes to the formation of thin cell walls and uniform cell morphology at high temperatures, indicating a good stretchability of the polymer melt that prevents cell wall rupture and gas loss. Strain hardening was the least pronounced in Resin C, and resulted in low volume expansion and thick cell walls, as the lack of extensional hardening makes it challenging for the stretched chains to capture and stabilize growing cells at elevated temperatures without rupture. On the other hand, the *T*_c,onset_ of the resins was found to be the deciding factor on how far below the melting point of the polymer foaming can be induced. While the volume expansion of all three resins diminished gradually as *T*_foaming_ approached *T*_c,onset_, the relatively lower *T*_c,onset_ of Resin A allowed sufficient expansion to occur at 120 °C—a phenomenon that was absent in the other two resins studied. Overall, by introducing a temperature protocol that is different from conventional batch foaming, this study successfully demonstrated that completely melting the polymer far beyond its melting point and initiating foaming to sufficiently low temperatures above the onset of crystallization provides a more accurate assessment on the foamability of semi-crystalline polymers, especially when it comes to continuous foam processes due to the close resemblance to the thermal history that the polymer undergoes: cooling upon complete melting. Most importantly, the work presented here offers further research directions in terms of the accurate assessment of the foamability of newly developed resins. For instance, semi-crystalline polymers with stronger extensional hardening and/or a higher degree of branching may be tested to verify if the high expansion achieved at high temperatures observed in this work holds true. Other highly desirable research directions may include a direct comparison of the foaming behavior of polymers subjected to batch and continuous foam processes under similar temperature histories and the amount of dissolved gas to investigate the effect of flow on foamability.

## Figures and Tables

**Table 1:** Summary of material properties of the PP homopolymers used for foaming. Melt strength and molecular properties were measured based on the ExxonMobil test method.

	Resin A	Resin B	Resin C
Melt Strength (190 °C) [cN]	58	45	40
Weight Average Molecular Weight [kg/mol]	585	541	520
Polydispersity Index	8.7	~13	~13

**Table 2:** Summary of the foamability of three resins subjected to two different batch foaming processes. (Note: * foaming temperature range was determined from conditions that led to Φ > 10; ** cell density range was obtained over the temperature range that led to uniform cell morphology). Units of cell density correspond to number (#) of nucleated bubbles per volume of foamed polymer.

Resin	*T*_c,onset_ [°C]	SHR	Method	Maximum Φ	Foaming Temperature Range * [°C]	Cell Density Range ** [#/cm^3^]
A	112	13	1	48 ± 2	150–175	10^7^–10^8^
			2	45 ± 3	120–170	10^7^–10^8^
B	117	5.6	1	27 ± 2	155–165	10^6^–10^7^
			2	36 ± 3	130–160	10^6^–10^7^
C	118	1.0	1	32 ± 4	160	10^6^–10^7^
			2	28 ± 4	130–150	10^6^–10^7^

## Data Availability

The data presented in this study are available on request from the corresponding author.

## Supplementary Materials

The following are available online at https://www.mdpi.com/article/10.3390/polym14010044/s1, Figure S1: Photograph of Resin B foamed at temperatures above its end melting point under Method 1 (top) and Method 2 (bottom), Figure S2: Cell size distribution of (a) Resin A, (b) Resin B, and (c) Resin C foams produced using Method 1 (i.e., partial melting of PP). End melting temperatures of the resins are 154 °C, 159 °C, and 165 °C, respectively.
