# Supplementary material for: Tuning High and Low Temperature Foaming Behavior of Linear and Long-Chain Branched Polypropylene via Partial and Complete Melting
Source: Polymers (Basel). 2021 Dec 23;14(1):44. doi: 10.3390/polym14010044 (PMC8747320; doi:10.3390/polym14010044)
Supplement: Supplementary file 1 [file polymers-14-00044-s001.zip › polymers-1479621-supplementary.pdf]

polymers

Supplementary materials

Tuning High and Low Temperature Foaming Behavior of Linear and Long-Chain Branched Polypropylene via Partial and Complete Melting

Mu Sung Kweon [1,†], Mahmoud Embabi [1,†], Maksim E. Shivokhin [2], Anvit Gupta [2], Xuejia Yan [2], George Pehlert [2] and Patrick C. Lee [1,*]

[1] Multifunctional Composites Manufacturing Laboratory (MCML), Department of Mechanical and Industrial Engineering, University of Toronto, 5 King's College Road, Toronto, ON M5S 3G8, Canada; mkweon@mie.utoronto.ca (M.S.K.); embabi@mie.utoronto.ca (M.E.)
[2] ExxonMobil Chemical Company, 5200 Bayway Drive, Baytown, TX 77520, USA; maksim.e.shivokhin@exxonmobil.com (M.E.S.); anvit.gupta@exxonmobil.com (A.G.); xuejia.yan@exxonmobil.com (X.Y.); george.j.pehlert@exxonmobil.com (G.P.)
* Correspondence: patricklee@mie.utoronto.ca; Tel.: +1-(416)-946-5407
† These authors contributed equally to this work.

Citation: Kweon, M.S.; Embabi, M.; Shivokhin, M.E.; Gupta, A.; Yan, X.; Pehlert, G.; Lee, P.C. Tuning High and Low Temperature Foaming Behavior of Linear and Long-Chain Branched Polypropylene via Partial and Complete Melting. *Polymers* **2022**, *14*, 44. https://doi.org/10.3390/polym14010044

Academic Editor(s): Markus Gahleitner

Received: 10 November 2021
Accepted: 20 December 2021
Published: 23 December 2021

Publisher's Note: MDPI stays neutral with regard to jurisdictional claims in published maps and institutional affiliations.

Copyright: © 2021 by the authors. Licensee MDPI, Basel, Switzerland. This article is an open access article distributed under the terms and conditions of the Creative Commons Attribution (CC BY) license (https://creativecommons.org/licenses/by/4.0/).

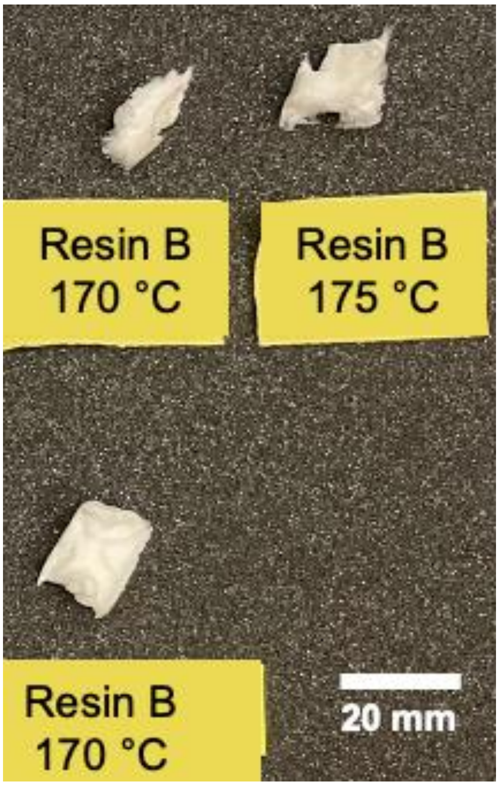

Figure S1. Photograph of Resin B foamed at temperatures above its end melting point under Method 1 (top) and Method 2 (bottom).

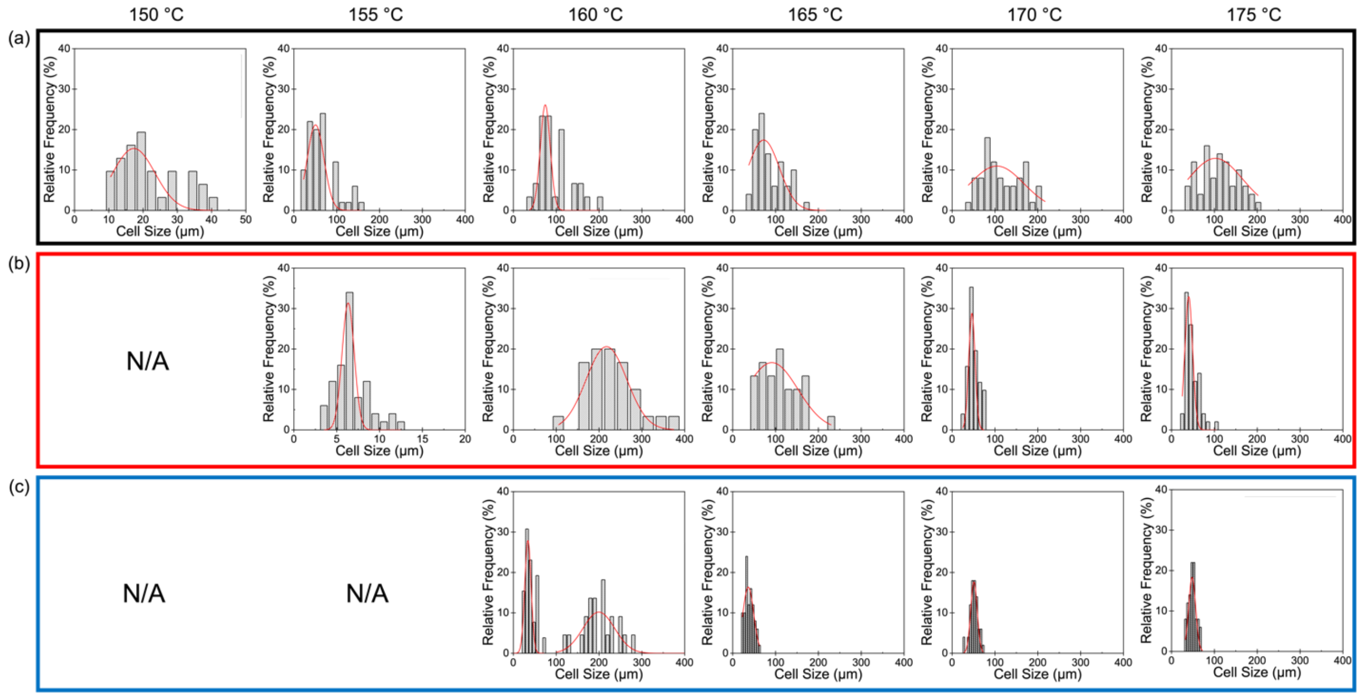

Figure S2. Cell size distribution of (a) Resin A, (b) Resin B, and (c) Resin C foams produced using Method 1 (i.e., partial melting of PP). End melting temperatures of the resins are 154 °C, 159 °C, and 165°C, respectively.
